# A method for the mix design of low carbon concrete towards industrial production

**DOI:** 10.1617/s11527-022-02040-5

**Published:** 2022-09-29

**Authors:** Federica Boscaro, Robert J. Flatt

**Affiliations:** 1 Institute for Building Materials, ETH Zurich, Stefano-Franscini-Platz 3, 8093 Zurich, Switzerland; 2 Department of Chemical Engineering, University of California, Santa Barbara, CA 93106 USA

**Keywords:** Blended cement, Hydration, Compressive strength, Rheology, Upscaling

## Abstract

The introduction of newly developed blended cements into the mass market is essential to ensure an effective reduction of the carbon footprint related to cement production. To facilitate this process, formulating mix proportions using pastes and/or mortars rather than concrete can be a great advantage. However, for the upscaling towards industrial concrete it is then essential to maintain the target rheological and mechanical properties, something that is all too often challenging. In this work, a procedure facilitating such an upscaling was illustrated in the form of a flow chart. Specifically, best practices to obtain a good correlation between concrete prepared in a laboratory and one prepared in a plant were presented. This includes new data showing how to accommodate for possible differences in temperature and/or water content between both situations. The dataset of state-of-the-art correlations between mechanical performance and heat of hydration, considering w/b ratios relevant to practice, were expanded. This greatly facilitates the mix design of concrete with particularly low clinker contents, which in this work were illustrated with a blended cement containing only 50% clinker.

**Supplementary Information:**

The online version contains supplementary material available at 10.1617/s11527-022-02040-5.

## Introduction

The use of low clinker cements is considered as one of the most effective strategies to reduce the carbon footprint associated with cement production [1]. Given that Portland cement manufacture is responsible for 5–8% of anthropogenic CO_2_ emissions [2], transferring such new technologies from the laboratory to the industrial scale has become paramount. However, despite the increasing use of Supplementary Cementitious Materials (SCMs) and the development of alternative binders [1, 3–5], concrete production involving low clinker cements is not as widely spread as it should be. Alkali-activated binders and calcined clay limestone cements (LC^3^) technologies represent some of the few exceptions, with industrial-scale or pilot concrete productions [5–9].

In all cases, large scale tests using concrete prepared in a plant are ultimately necessary to ensure the robustness of new concrete mix designs. This can however be more problematic than suspected since mechanical and rheological performances can be affected by factors as:—the type of mixer and/or its energy,—the moisture of sand and aggregates,—temperature,—accuracy of water dosing (including possible washing water remaining in the mixer) and—possible residues in the mixer from previous concrete productions. In particular, it is well known that mixing energy affects the hydration and rheology of cement and its constituents [10–12]. Further, while routine laboratory trials often involve dried aggregates, the contrary holds in practice. Similarly, the ambient and curing temperature may vary considerably, affecting cement hydration kinetics substantially [13–15].

Moreover, the simultaneous use of several admixtures is common practice nowadays, especially for low clinker cements, and may lead to competitive adsorption processes that affect the targeted rheological performance [16–19]. For instance, the carboxylic groups of polycarboxylate ether (PCE) superplasticizers compete with the ions of common activators of cement hydration (such as hydroxides and sulfates) on being adsorbed onto cement and SCMs particles, thus affecting the flow properties [16, 17]. However, this can be tailored by using compatible PCE molecular structures [18, 19]. Other than activators, PCEs show competitive adsorption with several types of chemical admixtures [20–22], however it is beyond the scope of this work to review the subject.

Considering all these factors, a proper predictability of rheological and mechanical performance from lab tests to plant production is called for.

An important issue here is to be able to account for performance changes in case the water content differs in industrial production. In this sense, correlations between calorimetry and strength of cement pastes or mortars are useful [23–28]. Specifically, the work of Bentz et al. demonstrates that compressive strength of mortars can be well correlated to the heat release measured by isothermal calorimetry, provided the heat is normalized with respect to the volume of liquid used [23]. They illustrated this for a variety of cement compositions and fineness, SO_3_ content, sand volume fraction and curing conditions (sealed and soaked), as well as for water to binder (w/b) ratios going from about 0.3 to 0.43. Above w/b of 0.43 they found that the relation becomes dependent on w/b, but did not investigate this feature further. One of the contributions of the present paper is to expand the dataset of such correlations between compressive strength and w/b towards higher w/b values. Such situations are indeed highly relevant to practice as they correspond to the largest volumes of concrete used and therefore also to the largest possible use of low clinker cements. Results presented in this paper also shed new light onto the underlying reason why strength correlates with the heat release per unit volume of water as reported by Bentz et al. [23].

Another important part of this paper is to establish that the relation between compressive strength and heat released does not depend on temperature. Thus, while hydration kinetics are temperature dependent, at equivalent cumulative heat release the same compressive strengths are obtained. This also represents a very useful result allowing to better anticipate how variability in production conditions may affect concrete properties in practice.

Based on these results a mix design method, presented in the form of a flow chart in Sect. 5.4, was proposed. It is a framework that aims at facilitating the optimization of low clinker cements and concrete at realistic w/b ratios and in presence of several chemical admixtures, from cement pastes to mortar, lab concrete and plant trials, in terms of target rheological and mechanical properties. It is emphasized that the role of paste volume was not considered. This factor profoundly affects the rheology of concrete, but has little effect on most hardened state properties as shown in the broad review compilation by Hermida [29, 30]. So, this work considered paste volume in mortars and concrete as defined by other considerations and focused on how the mix design optimization of a low clinker binder and related chemical admixtures may be done efficiently, ultimately proposing a framework for this.

Before this and in the next section, recent work on the correlation between rheological and mechanical performance of a new binder and a commercial one prepared either in the laboratory or in a concrete plant were summarized [31].

## Correlations of rheological and mechanical properties of concrete prepared in the laboratory and in a concrete plant

Recently, Boscaro et al. established correlations between fresh and hardened properties of concrete prepared in the laboratory (maximum 35 L) and in industry (maximum 3 m^3^) [31]. They tested two binders:

- a new low clinker blended cement with 50% Portland cement, 20% limestone, 20% burnt oil shale and 10% fly ash and activated by gypsum (abbreviated MF);
- a blend of a CEM II/A-LL 42.5 N (Fluvio 4, Holcim) and an SCM blend (Fluxolent, Holcim Switzerland) (abbreviated F4F, see Sect. 3.1 for details).

**Fig. 1:**
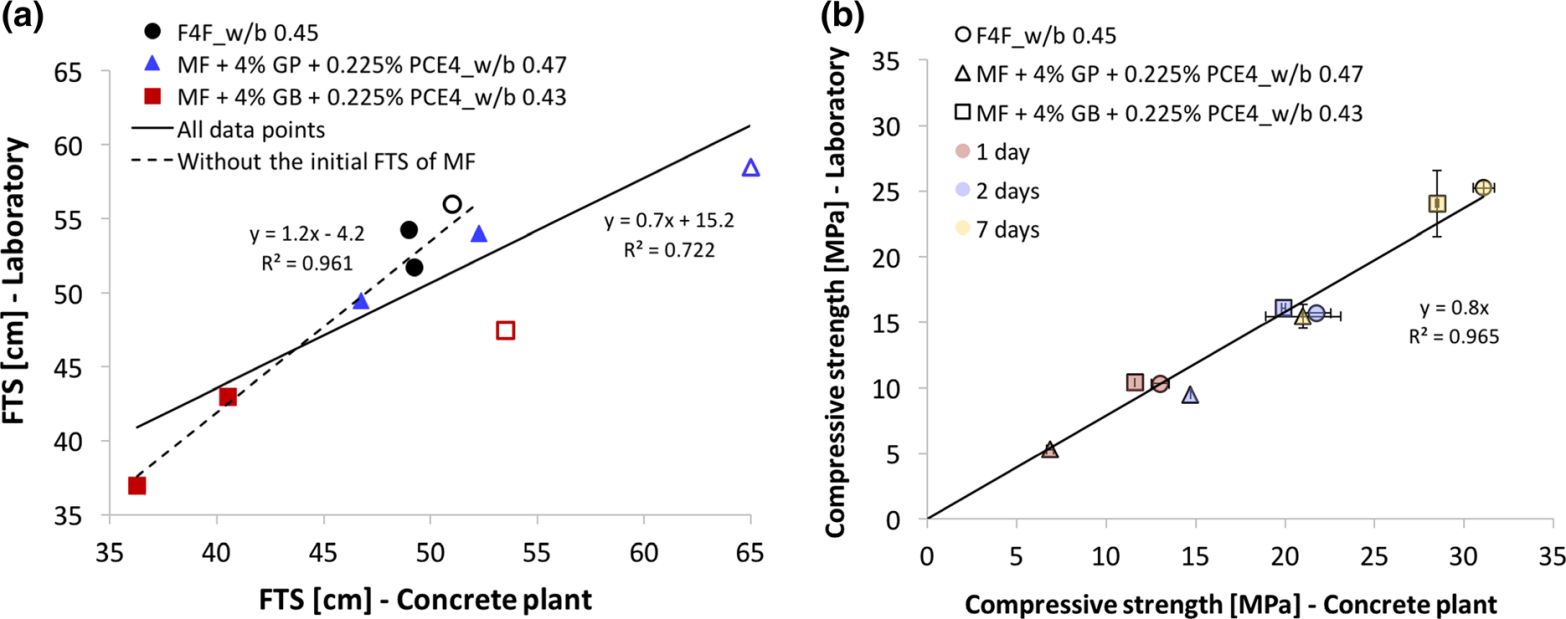
Correlations of flow and strength between lab and industry concrete. **a** Flow table spread. The open symbols represent the values measured at 10 min. **b** Compressive strength. GP indicates gypsum added as a paste, whereas GB when it is included as a powder. Adapted with permission from [31]

They found correlations between lab and industry flow table spread (FTS) from 10 to 90 min and for compressive strength from 1 to 7 days (Fig. 1). With regard to the FTS, values at 10 min were substantially higher for the MF industry concrete than for the lab one.

The same study also showed that temperature variations between 8 and 21 °C had no effect on the initial spread flow of cement pastes prepared with the MF binder and similar results were obtained by Boscaro for cement pastes containing the F4F binder [32]. This led to conclude that a change of the initial specific surface area of the hydrates in the MF binder is at the origin of the FTS difference between lab and industry concrete, which in turn may be due to different mixing energies and aggregates humidity between both situations [31].

Concerning compressive strength, Fig. 1b shows that all data from 1 to 7 days are well fitted by a common regression line for both binders. The slope obtained implies that lab concrete has compressive strengths 20% lower than the industry one, when considering the same curing time and w/b [31].

With regard to Fig. 1, it is essential to underline that the plant always had inaccuracies in the dosing of water. This was addressed by determining a posteriori the amount of water by the microwave method for each batch of concrete [31]. The origin of the errors in dosing are not all clear, but certainly involved residues from cleaning. To account for this, the laboratory results were obtained using the a posteriori determined amount of water. This is certainly not an efficient solution for regular practice, but here serves the purpose of establishing the existence of lab to industry correlations at the same water content. While the compressive strength is rather robust to the w/b inaccuracies (see Fig. S1), the fluidity is much more sensitive to such changes.

The above results have two important implications. First, poor correlations between lab and industry, in particular for fluidity, may often be linked to inaccuracies in the water content. Second, variations in the water content are an industrial reality that should be accommodate for without having to repeat all lab experiments after the industrial production has taken place. This motivates the first major point of the present paper, which is to expand the previously proposed relation between strength and heat of hydration to account both for w/b and temperature.

## Materials and methods

### Materials

A new blended cement (here abbreviated as MF) containing 50% CEM I 52.5R (OPC, LafargeHolcim), 20% burnt oil shale (BOS), 20% limestone (LL) and 10% fly ash (FA) [32] and a commercial CEM II/A-LL 42.5 N (Fluvio 4, Holcim) were used. The latter was admixed with a commercial finely ground concrete addition, composed of granulated blast furnace slag (GBFS), limestone and BOS (Fluxolent, Holcim Switzerland). The combination of the two is here referred as F4F.

 Table 1 shows the chemical composition of the powders measured by X-Ray fluorescence, the median diameter D_V50_ and the specific surface area SSA_BET_ measured as described in [32]. The mineralogical composition of the powders is reported in Table S1 (see Supplementary Material) and can be found in [32].

**Table 1:** Chemical composition (%w/w), D_V50_ (μm) and SSA_BET_ (m^2^/g) of OPC, BOS, LL, FA, Fluvio 4 and Fluxolent

	OPC	BOS	LL	FA	Fluvio 4	Fluxolent
CaO	62.8	28.69	53.76	6.55	62.94	39.79
SiO_2_	20.2	34.2	1.9	48.73	16.89	28.82
Al_2_O_3_	4.7	10.74	0.92	23.50	3.96	8.58
Fe_2_O_3_	3.1	6.65	0.4	10.59	2.48	2.4
MgO	2.1	1.9	0.35	1.54	1.92	4.63
MnO	–	0.1	–	0.1	–	–
TiO_2_	–	0.57	0.06	1.16	–	–
P_2_O_5_	0.19	0.29	–	0.48	0.3	0.08
K_2_O	1.03	2.0	0.06	2.5	0.77	1.01
Na_2_O	0.22	0.19	–	0.48	0.25	0.27
SO_3_	3.5	9.6	–	0.67	2.9	3.31
L.O.I	2.16	5.0	42.5	3.7	7.6	11.11
D_V50_	11.7	6.06	7.4	14.4	14.5	11.8
SSA_BET_	1.01	5.53	2.19	1.50	1.08	2.79

The aggregates (0–16 mm, from a natural limestone quarry, BSL Loruens) were composed of calcite (60–70%w/w), dolomite (10–15%w/w), quartz (5–10%w/w), feldspar (0–5%w/w, mainly plagioclase) and sheet silicates (5–10%w/w, mainly mica, some rare clays). The water absorption coefficient (WA24) of the aggregates was 0.8%, according to EN 1097–6. The granulometry is reported in Fig. S2 (see Supplementary Material).

Commercial ground gypsum (99% purity, FG200, Saint-Gobain Formula), characterized by D_V50_ of 16.73 μm and SSA_BET_ of 0.77 m^2^/g, was used as activator of concrete prepared with the MF binder.

A commercial superplasticizer (SP) Sika® ViscoCrete®-5063 T (Sika Technology AG) was used for concrete prepared with the F4F binder. PCE4, a non-commercial polycarboxylate ether superplasticizer (Sika Technology AG) containing defoamer and biocide, was included in the concrete prepared with the MF binder. PCE4 is a methacrylic-based polymer synthetized by esterification. Its molecular parameters are reported in Table 2. The dosage of PCE4 is expressed as % of active polymer by weight of binder (bwb).

**Table 2:** Molecular parameters of PCE4. C/E is the carboxylic functions per side chain, n is the number of repeating units, N the number of monomers in the backbone per repeat unit (B), P the number of monomers in one side chain (SC), K*_A,1_ the adsorption equilibrium constant calculated according to [18]

C/E	B M_w_ (g/mol)	SC M_w_ (g/mol)	*n*	*N*	*P*	K*_A,1_
2.6	5250	1000	17	3.6	23	67

A commercial air entraining agent (AEA) Sika® Luftporenbildner LPS A-94 (Sika Technology AG) was used in all concrete formulations.

### Methods

#### Sample preparation

Concrete was prepared using deionized water and 0–16 mm dried aggregates, using several w/b ratios ( Table 3). The binder and the aggregates were homogenized for 1 min at 60 rpm. Once the liquid was added, concrete was mixed at 60 rpm: F4F concrete, for 2 min (w/b of 0.48) or 2.3 min (w/b of 0.45); MF concrete prepared without gypsum (MF_I, MF_II) or activated by gypsum blended (MF_GB), for 2.4 min; MF concrete activated by gypsum paste (MF_GP), for 2.7 min. Prior to fluidity loss measurements, concrete was mixed for an additional minute.

**Table 3:** Mix formulations for 1 m^3^ of concrete. The one of concrete containing the F4F binder and prepared at a w/b of 0.45, and the one of MF_GB and MF_GP, at w/b of 0.47, are reproduced from [31] with permission

	F4F	MF_I	MF_II	MF_GB	MF_GP
Sand 0–4 mm [kg/m^3^]	883^a^/886^b^	873	873	873	873
Gravel 4–8 mm [kg/m^3^]	340^a^/342^b^	336	336	336	336
Gravel 8–16 mm [kg/m^3^]	476^a^/477^b^	470	470	470	470
Total aggregates [kg/m^3^]	1699^a^/1705^b^	1679	1679	1679	1679
Binder [kg/m^3^]	323	387	387	387	387
Fluxolent [kg/m^3^]	80	–	–	–	–
Gypsum [% bwb]	–	–	–	4	4
PCE4 [% bwb]	–	0.225	0.25	0.225	0.225
Commercial SP [kg/m^3^]	2.75	–	–	–	–
AEA [kg/m^3^]	0.81	0.97	0.97	0.97	0.97
w/b [–]	0.45/0.48	0.43	0.43	0.43	0.43/0.47

^a^Amounts used in the concrete prepared at w/b of 0.45

^b^Amounts used in the concrete prepared at w/b of 0.48

Gypsum as activator was added on top of the binder and aggregates following two modes of addition:—added as a powder and homogenized for 1 min at 60 rpm;—added as a paste (prepared as described in [32]), followed by the remaining total liquid divided in one batch of only water and one including the PCE4 and the AEA.

#### Calorimetric measurements

Calorimetric measurements were performed at 10 °C and 23 °C using an isothermal calorimeter I-Cal 8000 HPC (Calmetrix, Arlington, MA, USA). Concrete was sieved under 4 mm prior to the beginning of the measurements. The tests started about 12 min after the beginning of the hydration. The first 30 min of the calorimetric data were not considered for all the measurements, with the exception of MF_I and MF_II samples where the first 1.97 h and 1.48 h were not evaluated.

#### Flow table spread 

The initial FTS and the fluidity loss at 1 h were measured according to SN EN 12 350–5 using a tronco-conic mould of 20 cm height × 20 cm bottom diameter × 13 cm smaller diameter. The flow table was humidified prior to testing. After the removal of the mould, the flow table was shocked 15 times and two perpendicular diameters were measured.

#### Compressive strength

Compressive strength was measured on concrete specimens (15 × 15 × 15 cm), according to EN 196. Samples were demoulded after 1 day and stored at 10 °C/100%RH or 20 °C/95%RH prior to testing. 2—3 cubes were tested per formulation.

## Results

### Effect of curing temperature 

#### Flow table spread

 Table 4 reports the effect of two temperatures on the FTS over 90 min of concrete prepared with each of the two binders. It shows that temperatures of 13 °C and 20 °C do not particularly affect the rheological properties, especially at 10 min.

**Table 4:** Effect of ambient temperature of 13 °C and 20 °C on the flow table spread (FTS) of concrete over 90 min

FTS (cm)	F4F 13 °C	F4F 20 °C	MF_GB 13 °C	MF_GB 20 °C	MF_GP 13 °C	MF_GP 20 °C
10 min	55.0	56.0	46.0	47.3	47.5	44.0
45 min	54.3	54.3	38.5	43.0	41.3	37.5
90 min	52.3	51.8	35.0	37.0	36.8	32.3

Data are reported for concrete prepared with binders F4F, MF_GB and MF_GP with w/b ratios of 0.45 and 0.43, respectively. Data for F4F and MF_GB, both at 20 °C, are reproduced from [31] with permission

#### Compressive strength

**Fig. 2:**
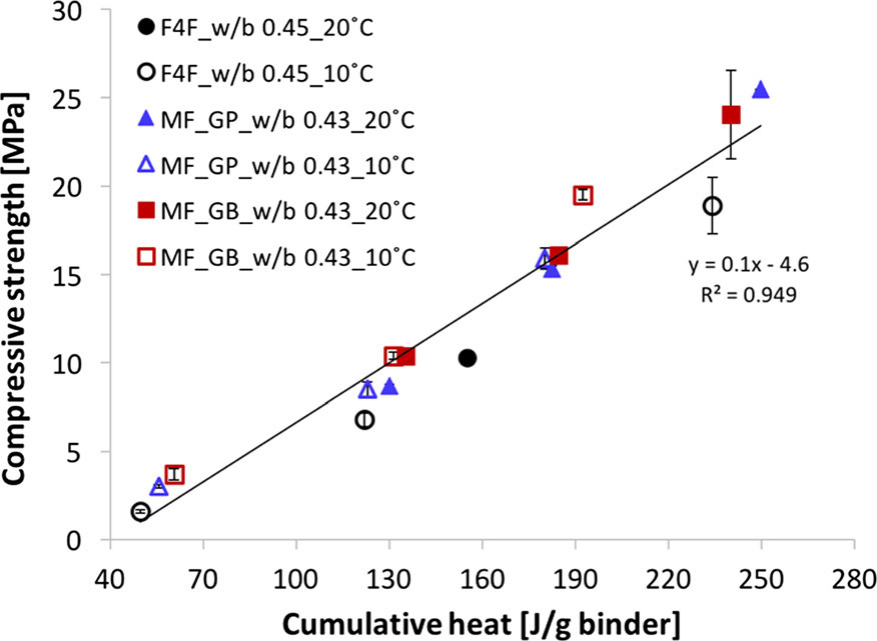
Effect of curing temperatures on the correlation between compressive strength of concrete and cumulative heat of concrete sieved to mortars at 1, 2 and 7 days. Samples were cured at 10 °C/100%RH and at 20 °C/95%RH. Data were collected on concrete prepared with binders F4F, MF_GB and MF_GP. Data for F4F and MF_GB, both at 20 °C, are reproduced from [31] with permission

 Figure 2 presents the compressive strength as function of the cumulative heat at 1, 2 and 7 days for concrete samples prepared with both binders and cured at 10 °C/100%RH and 20 °C/95%RH. All the data points are well fitted by a single regression line, which is not affected by the different curing temperature. So, while strength develops slower at low temperature, as confirmed in the data listed in Table 5, values are independent of temperature if the basis of comparison is a degree of hydration rather than a number of days after mixing. This finding is useful for low clinker concrete as further explained in Sect. 5.1.

**Table 5:** Compressive strength at 1, 2 and 7 days of concrete samples cured at 10 °C/100%RH and 20 °C/95%RH

Compressive strength [MPa]	F4F 10 °C	F4F 20 °C	MF_GB 10 °C	MF_GB 20 °C	MF_GP 10 °C	MF_GP 20 °C
1d	1.6 ± 0.1	10.3	3.7 ± 0.3	10.4	3.0 ± 0.1	8.7 ± 0.1
2d	6.8 ± 0.4	15.7	10.4 ± 0.2	16.1	8.5 ± 0.4	15.3 ± 0.2
7d	18.9 ± 1.6	25.3 ± 0.5	19.5 ± 0.3	24.1 ± 2.5	15.9 ± 0.6	25.5 ± 0.0

Data are reported for concrete prepared with the binders F4F, MF_GB and MF_GP. Data for F4F and MF_GB, both at 20 °C, are reproduced from [31] with permission

### Effect of the w/b on the strength to heat of hydration correlation

Following the approach proposed by Bentz et al. [23], the relation between compressive strength and heat of hydration expressed with respect to the initial water amount was examined. The data discussed were collected on three different types of concrete:

- with binder F4F, at w/b of 0.45 and 0.48;
- with binder MF
- without activator for w/b of 0.43 (noted MF_I and MF_II);
- with gypsum activator blended to the cement (MF_GB) for w/b 0.43
- with gypsum activator added as a paste (MF_GP) for w/b of 0.43 and 0.47.

The initial FTS of these mixes is reported in Table 4 and Table S2.

**Fig. 3:**
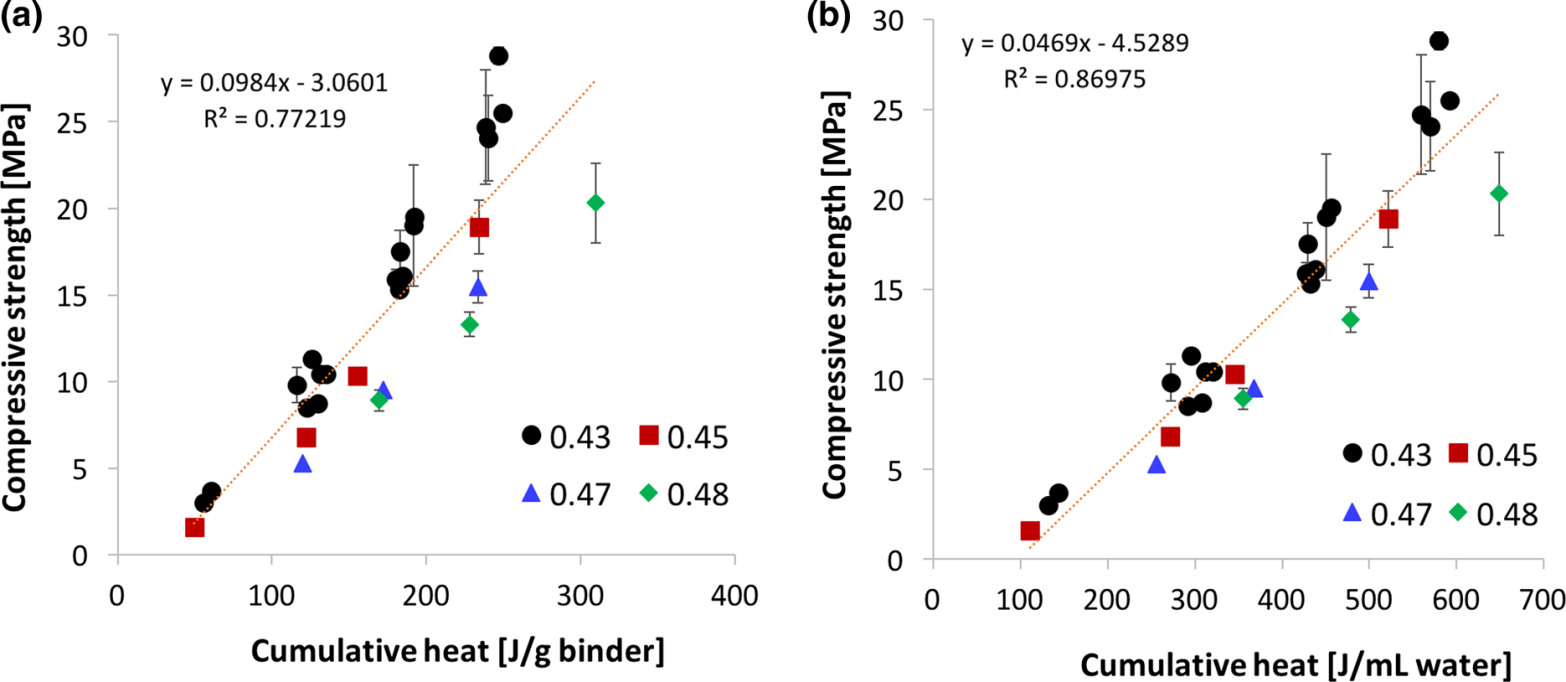
Effect of w/b ratios on the correlation between compressive strength of concrete from 1 to 7 days and the cumulative heat measured on concrete samples sieved to mortars. Data points are obtained on samples with binders F4F, MF_I, MF_II, MF_GB and MF_GP. **a** Cumulative heat is given per unit mass of binder. **b** Cumulative heat is given per volume of water. In both cases a single linear regression is plotted as a guide for the eye and the coefficient of correlation is given indicatively

 Figure 3a shows, as expected, that the relation between strength and cumulative heat depends on the w/b value, when the heat is given by mass of binder. This dependence on w/b is decreased, as suggested by Bentz et al. [23], when the cumulative heat is expressed with respect to the initial water content rather than with respect to the binder mass ( Fig. 3b). Data presented here lie above the w/b value of 0.43, which Bentz et al. identified as a value above which the strength to heat correlation depends on w/b [23]. This issue will be further discussed in the discussion section and a way to deal with it will be presented.

## Discussion

### Effect of temperature on FTS and on strength to heat correlation

It was shown that the temperature does not significantly affect the FTS of concrete from 10 to 90 min, confirming previous results by Boscaro et al. obtained on cement pastes [31]. This emphasizes the possibility that the higher initial FTS obtained by those authors at 10 min in the industrial concrete with the low clinker cement was probably related to differences in the initial surface area.

The correlation between compressive strength and cumulative heat, in particular for the MF binder, does not depend on temperature. However, the time needed to release a given amount of heat and thus to reach a given compressive strength does depend on temperature ( Fig. 2 and Table 5). The curing temperatures studied are 10 and 20 °C, which are among the ones investigated by Lothenbach et al., who reported small differences between the hydrate phase assemblage from 5 to 30 °C for a CEM II/A-LL 42.5R [15]. On the other hand, higher curing temperatures have been reported to negatively affect the compressive strength at later ages [14, 15]. In particular, Gallucci and Scrivener reported an increase in the apparent density of C-S–H from 5 to 60 °C, which results in a higher capillary porosity and consequently leads to a lower compressive strength [13]. A higher C-S–H density was also observed by Lothenbach et al., along with a reduction in the ettringite and calcium monocarboaluminate contents at 40 °C [15]. In contrast to those results, the narrower, but highly practice relevant temperature range considered here, does not affect the relation from degree of hydration to strength. This is good news as implies that a single temperature is needed to calibrate the relation between strength and temperature. Then by a simple calorimetry measurement at another temperature it is possible to predict the strength development at that other temperature. This statement is however based on results at 10 and 20 °C and would deserve further validation over a broader temperature range.

### Role of the w/b ratio

**Fig. 4:**
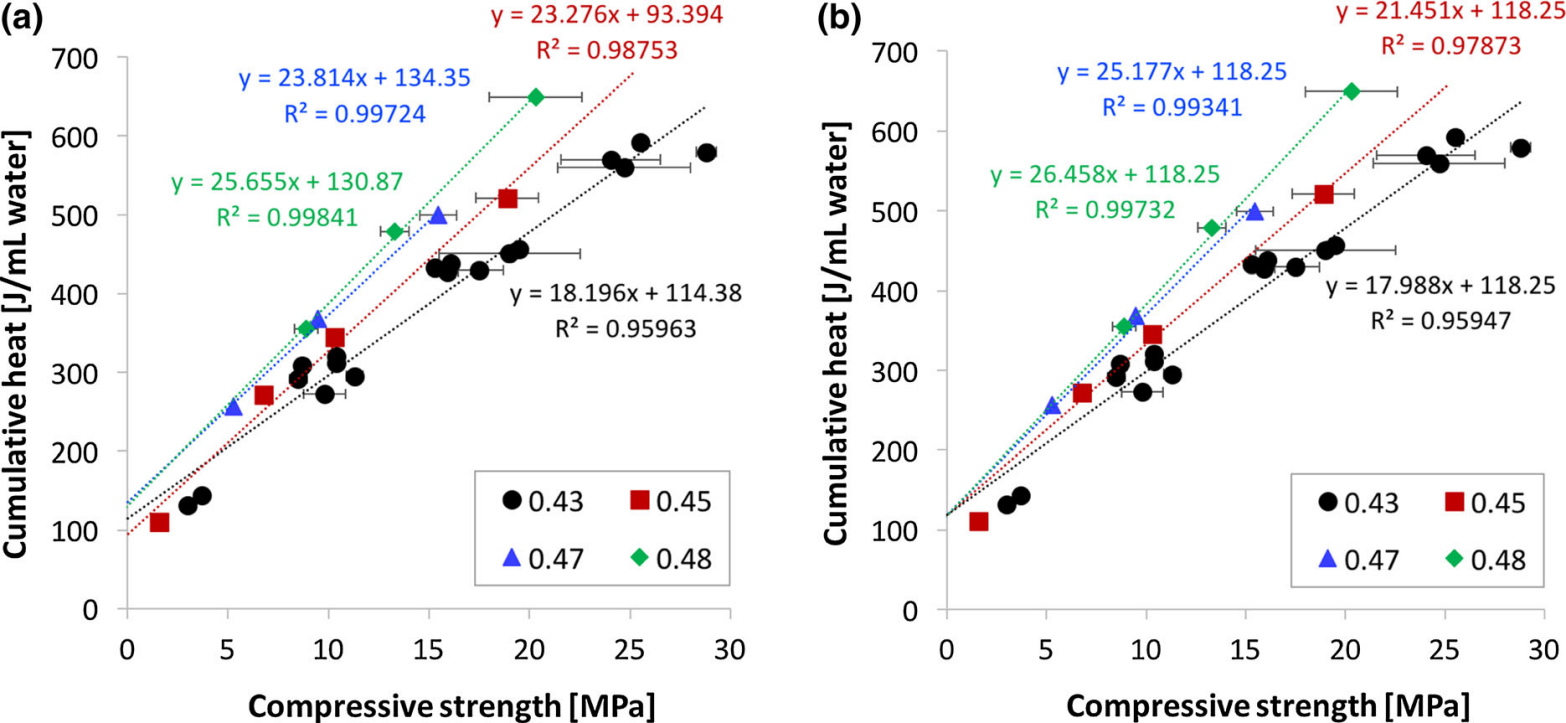
Effect of w/b ratios on the correlation between compressive strength of concrete from 1 to 7 days and the cumulative heat measured on concrete samples sieved to mortars. Data points are obtained on samples prepared with binders F4F, MF_I, MF_II, MF_GB and MF_GP. Cumulative heat is given per volume of water. The regression lines in a) are forced to an average ordinate in (b)

**Fig. 5:**
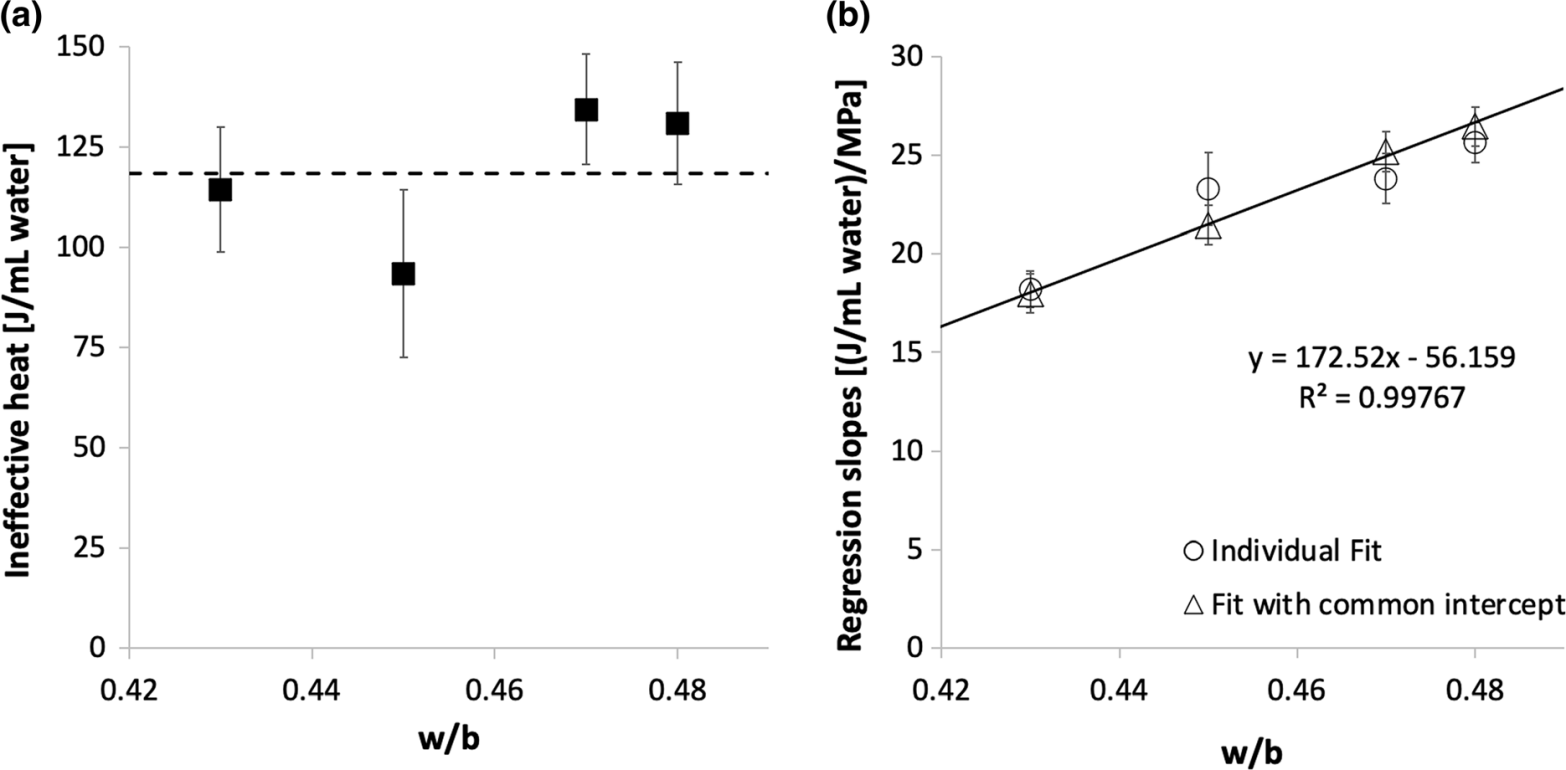
**a** Ineffective heat H_0_, obtained from regressions in Fig. 4a, plotted versus w/b. No dependence on w/b ratio is observed. **b** Regression coefficients from Fig. 4 versus w/b. The individual fit is obtained from Fig. 4a, while the one with the common intercept from Fig. 4b. Error bars represent the standard errors on the parameters obtained from the corresponding regressions

Figure 3 reflects the relation between the degree of hydration and the strength, that is how these data are most often represented in the literature. From Fig. 3b, it is apparent that a single linear regression does not account well for the role of w/b, even if the cumulative heat is reported with respect to the mass of water rather than binder. Also, the intercept for zero strength suggests the existence of a certain “ineffective heat”. To best examine this and because of the fitting procedure proposed in this section, it is suitable to plot heat versus compressive strength as in Fig. 4 (Fig. S3 shows this with heat per mass of binder). This plot underlines that the “ineffective heat” is very similar between all data series. In fact, as highlighted in Fig. 5a, where values are plotted versus w/b, the “ineffective heat” is independent of w/b. Consequently, the average value (118 J/mL as given by the discontinuous line in Fig. 5a) can be used to refit all data series, as shown in Fig. 4b.

The meaning of the “ineffective heat” is discussed in the next sub-section. For now, the slopes of the regressions from Fig. 4a, b is considered. As shown in Fig. 5b where they are reported in relation to w/b, values do not change much when using a common value for the “ineffective heat”. Moreover, these slopes can be very well fitted by a linear regression (continuous line in the same figure). Thus, cumulative heat *H* per water volume relates to compressive strength *Rc* through:

1 $$\mathrm{H}={\mathrm{H}}_{0}+\mathrm{Rc}\left(\mathrm{\alpha w}/\mathrm{b}+\upbeta \right)$$

where *H*_*0*_ is the ineffective heat (here 118 J/mL), *α* and *β* are respectively the slope and ordinate of the regression line in Fig. 5b (172.5 and -56.2 both in units of (J/mL water)/MPa).

The compressive strength can therefore be given in relation to w/b by:

2 $$\mathrm{Rc}=\frac{\mathrm{H}-{\mathrm{H}}_{0}}{\mathrm{\alpha w}/\mathrm{b}+\upbeta }$$

Interestingly, the same equation also provides a good fit if the cumulative heat is expressed per mass of binder, instead of per volume of initial water (see Fig. S4). In both cases, the value of *β* is negative, which implies that the relation leads to an infinite compressive strength for a finite and critical value of w/b. If the data are fitted in terms of heat per mass of binder that critical w/b is 0.362, while it is 0.326 otherwise (based on the regression in Fig. 5b).

**Fig. 6:**
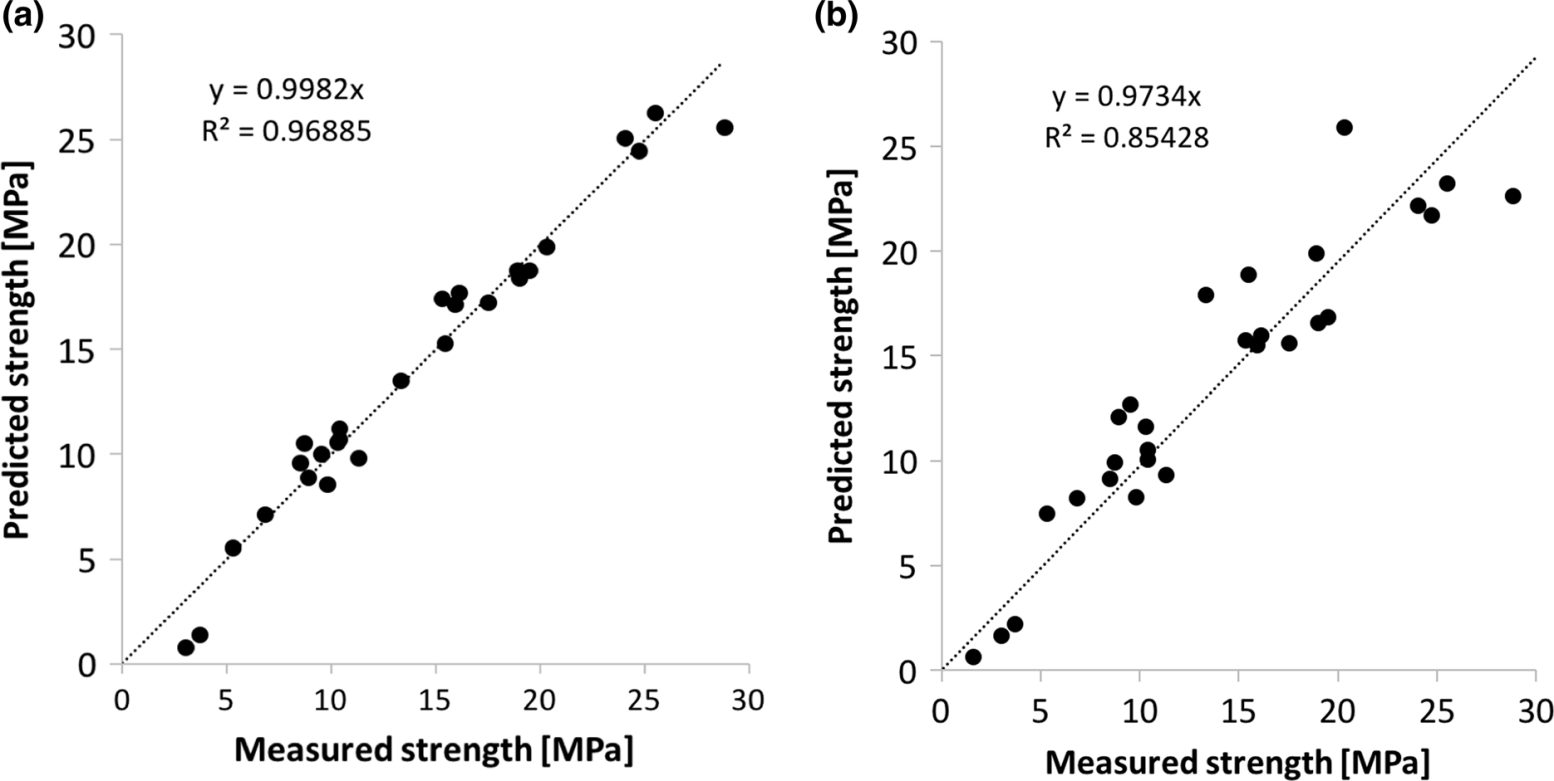
Comparison of measured and predicted compressive strength. **a** model defined in this paper, including a dependence on the w/b ratio, **b** model proposed by Bentz et al. [23] for w/b ratios lower than 0.43 and neglecting the dependence on the w/b

Using Eq. (2) with *H*_*0*_ = 118 J/mL, *α* = 172.5 J/mL and *β* = -56.2, the predicted compressive strengths versus the measured ones are reported in Fig. 6a. The fit is very good with a coefficient of correlation of 0.97. In contrast, if the role of w/b is neglected, the coefficient of correlation is substantially worse (0.85), as highlighted by the larger scatter in Fig. 6b. The improved model presented here includes an additional fitting parameter, which is justifiable owing to the excellent correlation in Fig. 5b.

The compressive strength can actually also be well fitted by Eq. (2) when the cumulative heat is expressed per mass of binder instead of per volume of initial water (Fig. S5). In fact, the same coefficient of correlation is obtained (0.97). However, owing to the very good fits obtained by Bentz et al. across a large number of mixes below w/b 0.43 [23], their suggestion of expressing the cumulative heat with respect to the volume of water is maintained.

### Meaning of the ineffective heat of hydration

As previously mentioned, the correlation of strength with cumulative heat of hydration has been reported by many researchers [23–28]. However, the work of Bentz et al. [23] stands out in that it proposes to report heat with respect to the amount of water rather than the cement mass. While that representation may be counter intuitive, it can tentatively be rationalized considering the filling of initial porosity.

The broad range of compositions over which the normalization with respect to the volume of water provided better correlations suggests that there is an underlying common mechanism to consider. This may be that the space filling of the volume initially occupied by water controls the strength development [23]. With respect to the “ineffective heat”, Bentz et al. argued that it corresponds to a volume of hydrates needed for percolation and that this is independent of w/b when w/b is below 0.43 [23]. On the contrary, they also argued that *H*_*0*_ should increase with w/b above that limit. Specifically, they reported this for a mix with w/b of 0.56 [23]. However, later on Lootens and Bentz identified a similar value of *H*_*0*_ for a CEM I 42.5 having w/c between 0.3 and 0.5 [28].

In the results presented here the value of *H*_*0*_ is 118 J/mL, rather similar but nevertheless different to the one of 180–200 J/mL reported by Bentz et al. [23] and Lootens and Bentz [28]. This may reflect that the binders studied in this work have a lower initial heat of hydration than those used by Bentz et al. [23] and Lootens and Bentz [28], but nevertheless do react at early age. Indeed, the mixed binders used by Bentz et al. [23] included limestone and fly ash, which do not react much at early ages. In contrast, the combination of SCMs used in this paper contributes to the heat release already before 48 h [32] and this most probably involves reactions that are less exothermic than the hydration of OPC. Thereby the volume of hydrates needed for percolation would be obtained for a lower value of the “ineffective heat”.

As first order analysis, an average ineffective heat of 150 J/mL between both studies is considered. The amount of C_3_S that must react to produce 150 J is about 0.28 g, thus about 0.10 cm^3^. Stated differently, it implies that about 10% of the initial porosity needs to be filled by the additional volume occupied by hydrates before substantial strength may develop. The amount of C_3_S that must dissolve is much higher than what is needed to saturate the pore solution (in the range of 2 mg), which adds weight to the percolation argument.

In relation to cement composition and correlation between early strength and heat of hydration, other authors showed that an extra 4% of C_3_A increases the heat release but does not affect the compressive strength at 1 day [25]. In contrast, they also reported that an increase of up to 1.0% of Na_2_O eq. increases the strength but does not significantly change the heat of hydration [25]. Most importantly, the same authors also reported that when the C_3_S content was increased, the compressive strength increased also [25]. This offers an indirect support to the argument that SCMs that are sufficiently reactive at early stages of hydration, may impact the value of *H*_*0*_.

### Planning and evaluation of the upscaling in new binder development

**Fig. 7:**
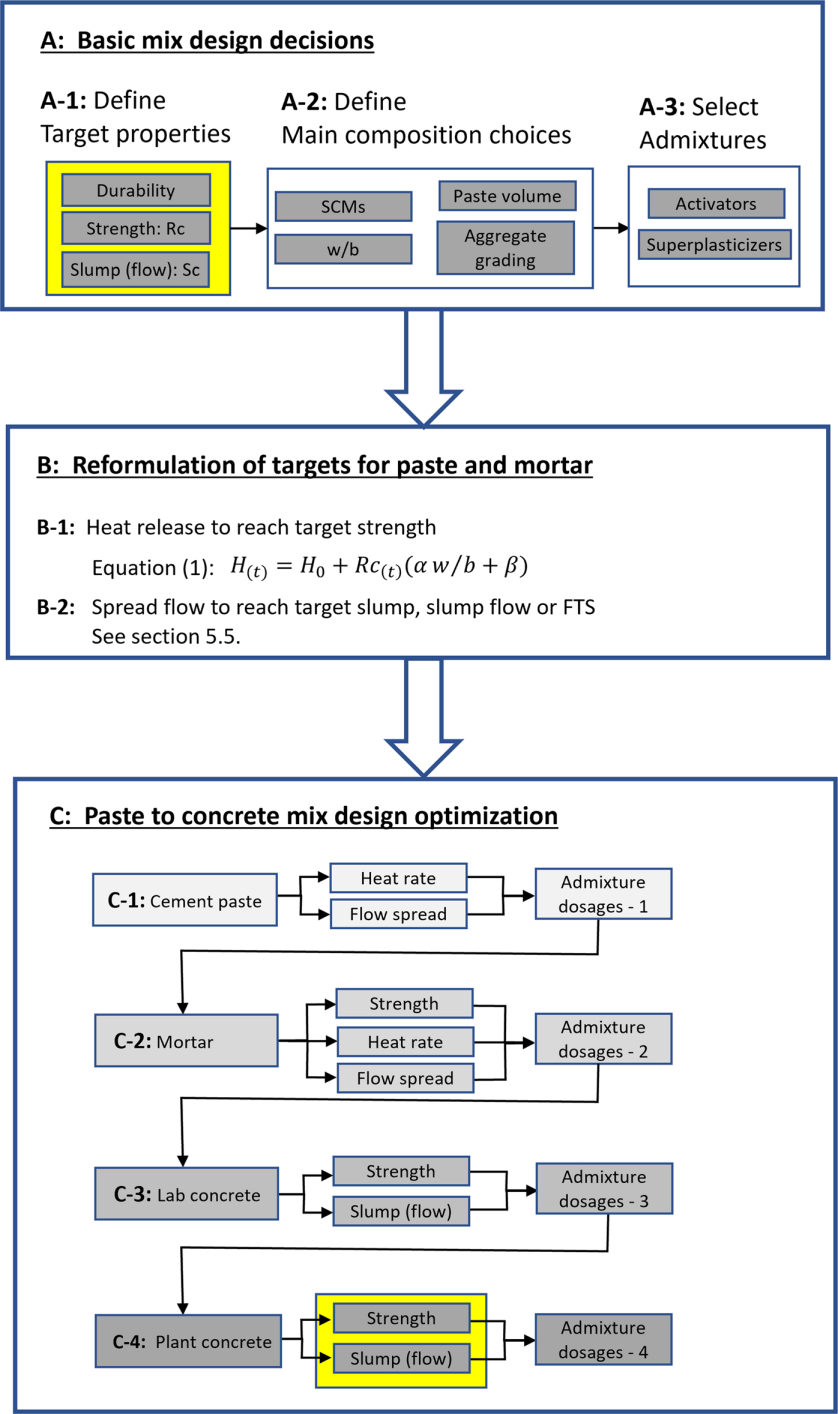
Flow chart for a reduced work-load strategy to optimize a low carbon concrete mix design. The first part A includes the main mix design choices. A-1 defines target properties of durability, strength and slump flow. A-2 defines key composition parameters, while A-3 selects the admixtures. The second part B consists in expressing the objectives defined in A as properties to be achieved on pastes and mortars. The third part C consists in optimizing the admixture compositions, possibly w/b if really needed, by successively working on the scales of paste, mortar, lab concrete and industrial concrete. The work-load decreases from step to step and delivers the final admixture dosages needed to achieve the goals defined in A

These results lead to the main contribution of this paper, which is to present a framework that encourages a more efficient optimization of low clinker cements and concrete to reach target properties for conventional construction. Such a strategy is presented in the flow chart in Fig. 7. It is emphasized that it targets low clinker concrete with extensive use of chemical admixtures and with realistic w/b ratios, for which the established mix design methods are not well applicable. However, it needs to be experimentally validated for a broad acceptance. This approach consists in the following steps:

 A. Basic mix design decisions:

1. Define the target concrete properties as the slump flow, compressive strength and durability. At this stage, considerations of durability serve to define a range of w/b values and are not considered further in the chart.
2. Define the main compositional parameters of the low carbon concrete: Type and amount of SCMs, paste volume and aggregate grading. Along with considerations on strength and durability, this largely determines the initial choice of the w/b, which is also decided at this stage.
3. Select chemical admixtures, particularly activators and superplasticizers.

B. Reformulate target properties in parameters to be achieved in paste and mortar:

1. From Eq. (1), determine the heat needed to reach the target compressive strength at 1, 3 and 7 days (or other selected times). The next steps involve an optimization from paste to concrete, mainly to adjust the dosage of these admixtures.
2. Using correlations between flow spread on paste (and mortars) and simple rheological tests in concrete (slump flow, FTS, etc.), determine the target flow spread to be obtained in pastes (and mortars). Procedures building on [19] are detailed in Sect. 5.5.

C. Optimize the mix design moving up from pastes to mortars and concrete:

1. Using flow spread measurements and calorimetry, optimize the accelerator and superplasticizer dosages to reach the target flow spread and heat release. For practical purposes, the onset of the acceleration period would most often not be wanted prior to 2 h after mixing.
2. In mortars, compressive strengths are measured, which along with calorimetry and flow spread measurements allow to refine the admixture dosages. Should the target properties be difficult to reach, modifications of the w/b and SCM proportions may be considered.
3. Experiments on laboratory concrete are conducted and offer the possibility to carry out minor adjustments of the admixture dosages to reach the target strength and slump flow. In principle, properties determined here should match the ones of concrete prepared in industry, provided the w/b is the same (see Sect. 2).
4. Ultimately, the optimized mix design resulting from the previous steps is tested on industrial concrete and final adjustments may be done to match the initially targeted properties.

Within this framework, the results presented in this paper and in particular Eq. (1) offer the means of defining the heat release needed to achieve prescribed strengths. This transfers the problem of mix design from the scale of concrete to that of pastes, since calorimetry measurements can replace compressive strength. This is particularly important because optimizing a mix design for low carbon concrete can be demanding, requiring in particularly many iterations of the superplasticizer and accelerator dosages, especially considering competitive adsorption may take place [16–19]. This can be very time consuming if only done in concrete.

The flow chart presented in this work specifically addresses this issue, by helping, for low clinker concrete prepared with several chemical admixtures, to maximize the mix design work that can be done using pastes and mortars rather than concrete. This entails substantial savings in time and thereby should facilitate a broader adoption of low carbon concrete with extensive use of admixtures in industry. However, it needs to be experimentally validated for a broad acceptance. As a side note, it is worth noting that many variables can be considered at the paste scale, so that use of statistical methods for the design of experiments would become the most effective procedure.

Finally, while results presented in this paper focused on the first 7 days owing to the isothermal calorimetry resolution, complimentary approaches for longer times may be used. Indeed, Bentz et al. showed that the increase in strength from 7 to 28 days could be predicted based on heat released during that period and measured by ASTM C186-05, which is a heat of solution method that does not suffer the long term resolution issues as isothermal calorimetry [23, 33]. It remains to be seen whether this also holds for slightly higher w/b and in particular if the w/b dependence identified in this work still holds. This norm has however been withdrawn on the basis of safety and useability issues. It thus does not represent a broadly useable solution, although a well-established lab would be expected to be able to conduct such experiments safely and reliably.

### Relating rheology from paste to concrete

While this paper focused on predicting strength, the relation from the fluidity of a paste to that of concrete remained elusive. It has in particular not dealt with the dependence of the rheology on the paste volume, which it was previously mentioned to be important, while only playing a secondary role in many hardened state properties (apart from creep) [29, 30]. Within the framework selected, it is assumed that relevant choices are made in terms of sand content in the mortar and aggregates in the concrete. With this in hand, changes at the paste level will lead to yield stress changes that should lead to corresponding relative changes of yield stress whether in mortars or concrete. Beyond this and regarding specific issues of upscaling, the following principle may be used. If a paste is mixed with an energy representative of what it would experience in concrete, then the yield stress of that paste should be proportional to that of the concrete. The proportionality constant depends on factors as the paste volume and grading of the sand and aggregates. As mentioned in [19], such a proportionality, as well as established relations between flow spread tests and yield stress, imply a proportionality between spread test results in concrete and paste. For the concrete discussed in this paper, a flow spread of 13 cm in pastes is a good starting point to reach a target FTS of 50 cm in concrete [19]. For other mix designs this relation would have to be adapted, with only a very limited number of experiments.

In terms of obtaining a first estimate of the superplasticizer dosage for a target fluidity, it is noted that for PCEs, correlations reported in [19, 34] allow this to be done in relation to the molecular structure, provided the flow spread of a paste containing a reference PCE of known structure is previously measured.

## Conclusions

A critical step for expanding the use of low carbon concrete into the mass market is to minimize the amount of work needed to obtain robust mix designs that deliver the prescribed fresh and hardened properties. In this sense, correlations between compressive strength and heat of hydration are very useful because they make it possible to carry out most of the mix design by using pastes instead of concrete.

In this regard, results presented in this paper expand the type of strength to heat of hydration relation proposed by Bentz et al. [23] to deal with w/b ratios of greater relevance for ordinary concrete. It was also shown that these relations are not temperature dependent.

Building upon this, this paper proposes a chart summarizing how to best use paste and mortar tests to formulate concrete mix designs that will perform as expected when prepared in industry. It is mainly intended for low clinker concrete that are prepared with chemical admixtures and at realistic w/b ratios. Such a workflow reduces the work-load for formulating well-working low carbon concrete mix designs and should therefore facilitate a broader use of such concrete in industry.

## Supplementary Information

Below is the link to the electronic supplementary material.

Supplementary file1 (DOCX 599 kb)
