# Supplementary material for: A method for the mix design of low carbon concrete towards industrial production
Source: Mater Struct. 2022 Sep 29;55(8):213. doi: 10.1617/s11527-022-02040-5 (PMC9522730; doi:10.1617/s11527-022-02040-5)
Supplement: Supplementary file 1 — Supplementary file1 (DOCX 599 kb) [file 11527_2022_2040_MOESM1_ESM.docx]

Materials and Structures

*Supplementary Information (SI)*

**A method for the mix design of low carbon concrete towards industrial production**

Federica Boscaro^1,2^, Robert J. Flatt^1^*

^1^Institute for Building Materials, ETH Zurich, Stefano-Franscini-Platz 3, 8093 Zürich, Switzerland

^2^Department of Chemical Engineering, University of California, Santa Barbara, CA 93106, USA

*Corresponding author. E-mail address: flattr@ethz.ch

| 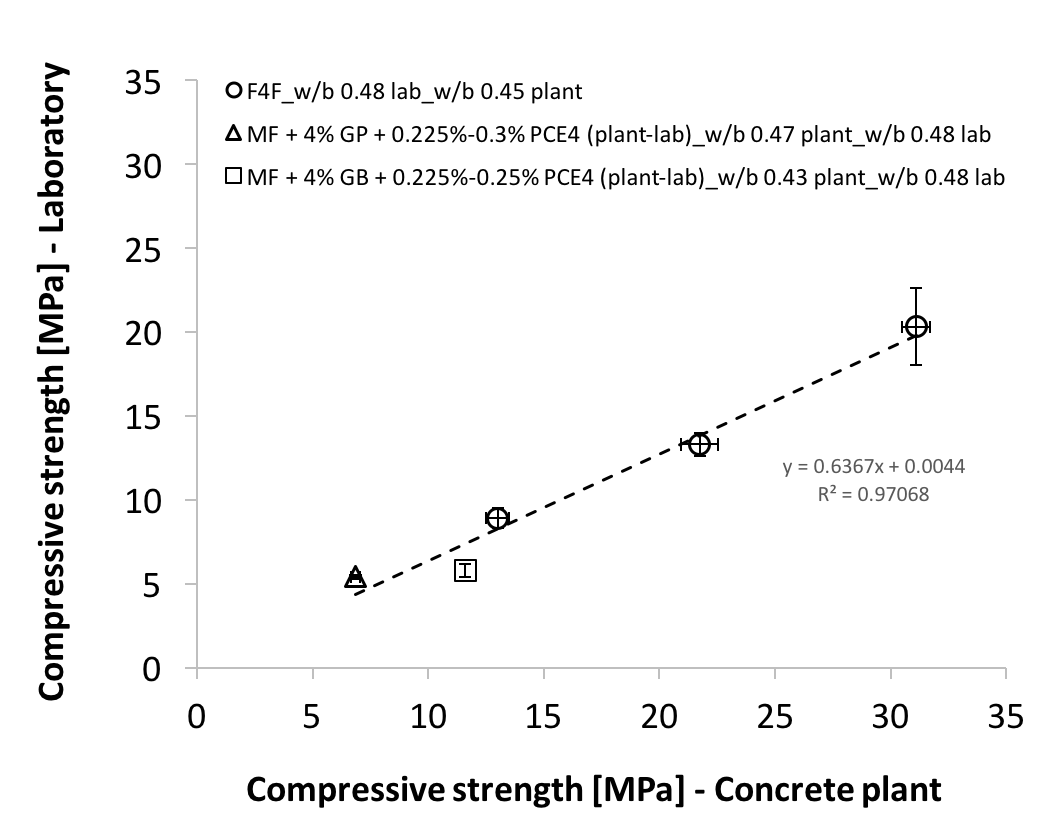 |
| --- |

***Fig. S1*** *Correlation between the compressive strength of concrete prepared in the laboratory and in a concrete plant. For the same formulation, the w/b ratio changes between the laboratory and the plant. On concrete prepared with the MF binder, the dosage of PCE4 is slightly modified, together with the w/b ratio, from the laboratory to the concrete plant. Data for the concrete plant are reproduced from [1] with permission*

***Table S1*** *Mineralogical composition (%w/w) of OPC, BOS, LL, FA, Fluvio 4 and Fluxolent as determined by Rietveld refinement of XRD measurements [2]*

|  | OPC | BOS | LL | FA | Fluvio 4 | Fluxolent |
| --- | --- | --- | --- | --- | --- | --- |
| Alite | 67.6 | - | - | - | 57.2 | - |
| Belite | 6.4 | - | - | - | 8.4 | - |
| C_3_A | 4.6 | - | - | - | 4.6 | - |
| C_4_AF | 8.7 | - | - | - | 7.3 | - |
| Gypsum | 4.6 | - | - | - | 3.7 | - |
| Anhydrite | - | 14.8 | - | - | - | 3.5 |
| Quartz | - | 16.2 | - | 1.1 | 0.2 | 4.0 |
| Calcite | - | 8.5 | 100 | - | 18.1 | 31.4 |
| Hematite | - | 3.5 | - | 1.1 | - | 0.4 |
| Illite | - | 15.4 | - | - | - | 0.4 |
| Portlandite | - | 0.7 | - | - | 0.3 | 3.4 |
| Free lime | 0.4 | 4.2 | - | - | 0.2 | 1.0 |
| Mullite |  | - | - | 3.6 | - | - |
| Lorenzite |  | - | - | 0.1 | - | - |
| Amorphous | 7.7 | 36.7 | - | 94.1 | - | 55.9 |

| ***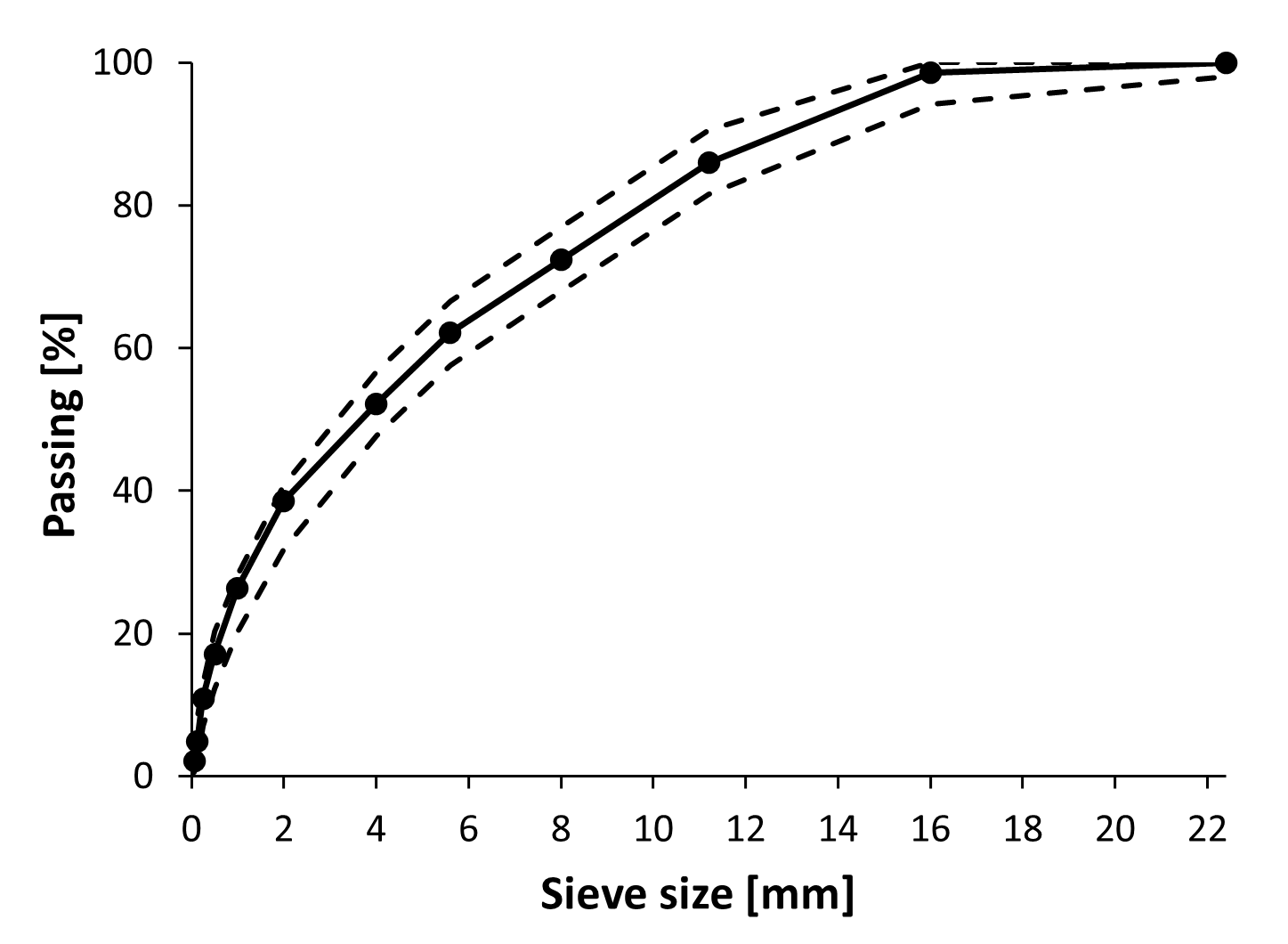*** |
| --- |

***Fig. S2*** *Granulometry of the aggregates according to EN 933-1. The dashed lines indicate the upper and lower limit passing, while the continuous line is the achieved passing*

***Table S2*** *Flow table spread (FTS) at 10 min of concrete prepared with the commercial CEM II/A-LL 42.5N admixed with Fluxolent (F4F) at a w/b of 0.48, the new low clinker cement without activator (MF_I, MF_II) and activated by the gypsum paste (MF_GP), at a w/b of respectively 0.43 and 0.47. The room temperature during concrete preparation corresponded to 20°C. The data for MF_GP is reproduced from [1] with permission*

| FTS [cm] | F4F  (w/b 0.48)  20°C | MF_I  (w/b 0.43)  20°C | MF_II  (w/b 0.43)  20°C | MF_GP  (w/b 0.47)  20°C |
| --- | --- | --- | --- | --- |
| 10 min | 58.5 | 45.6 | 51.8 | 58.5 |

a) b)

| 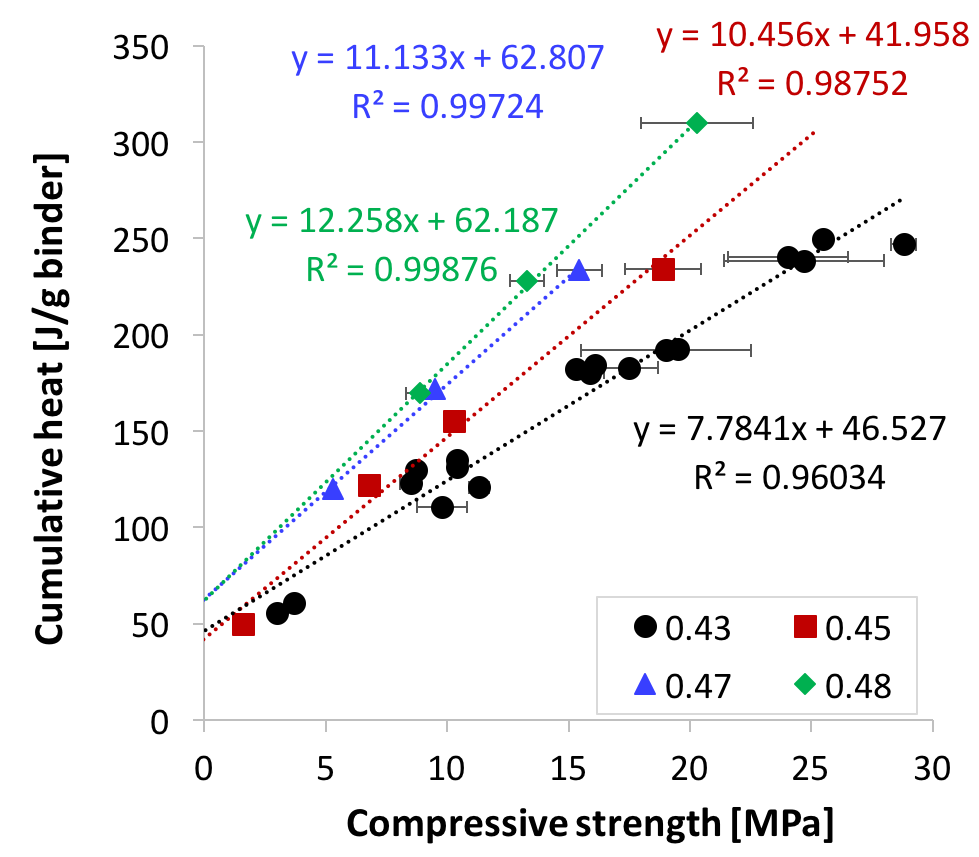 | 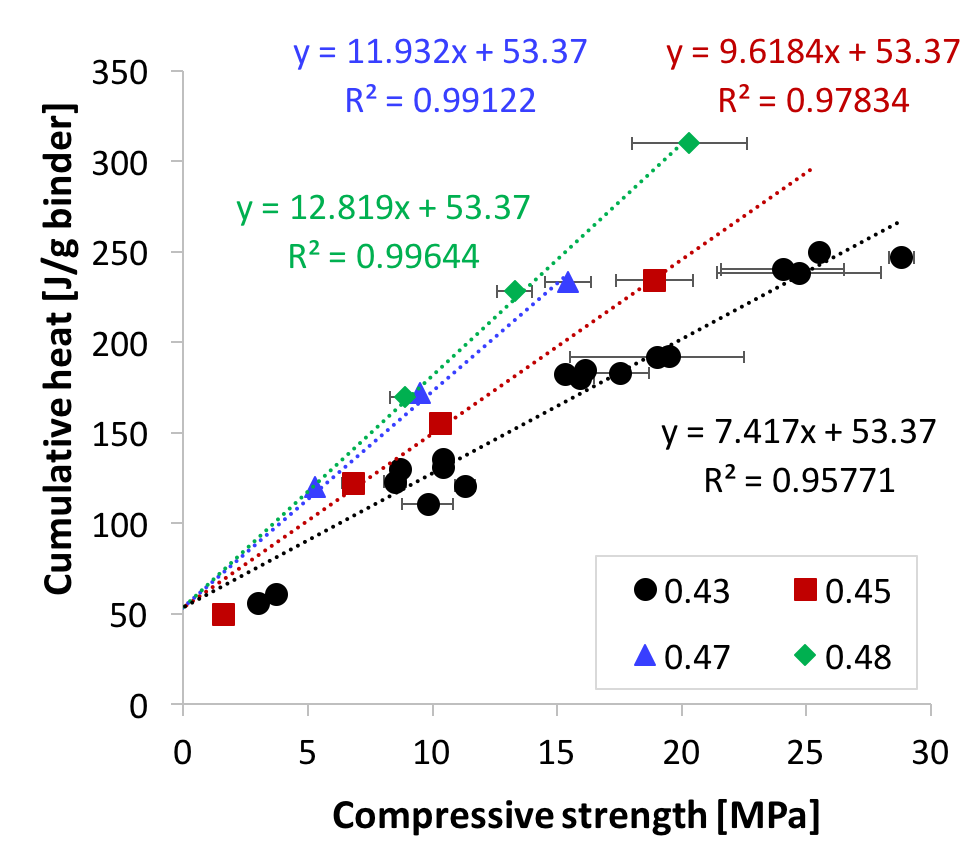 |
| --- | --- |

***Fig. S3*** *(corresponding to Fig. 4 in the paper) Effect of w/b ratios on the correlation between compressive strength of concrete from 1 to 7 days and the cumulative heat measured on concrete samples sieved to mortars. Cumulative heat is given per mass of binder. The average ordinate of each regression line in a) is used to force all the regression lines in b) [2]*

| 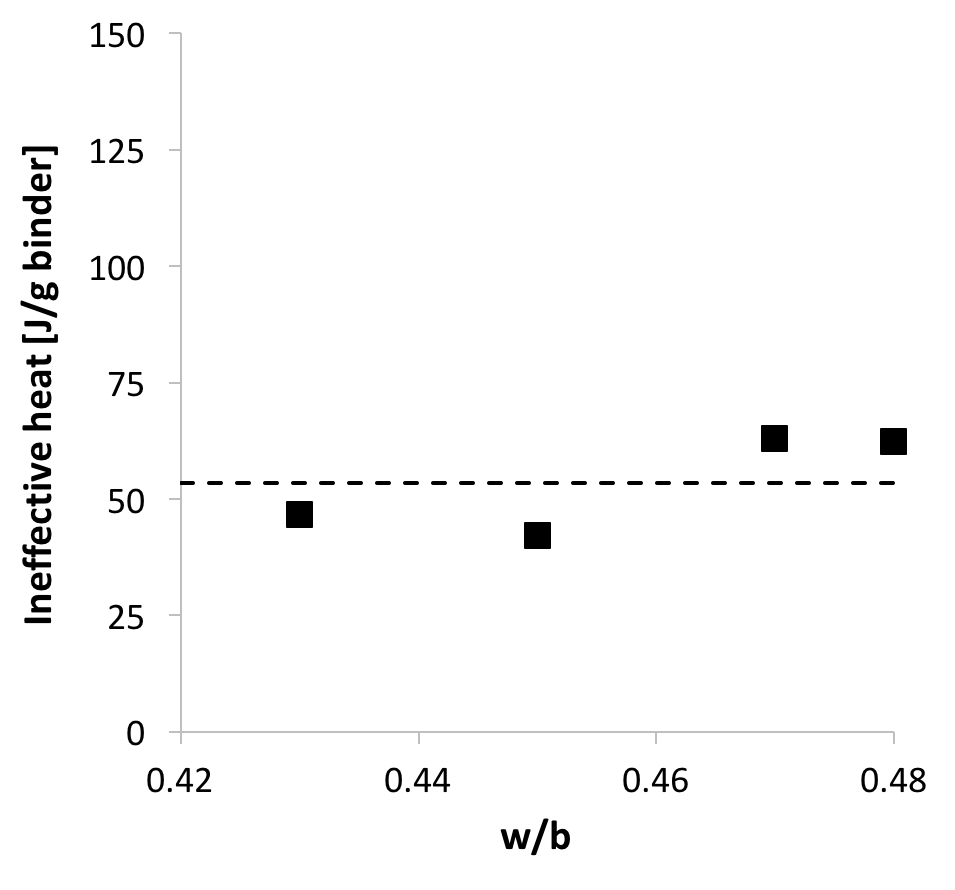 | 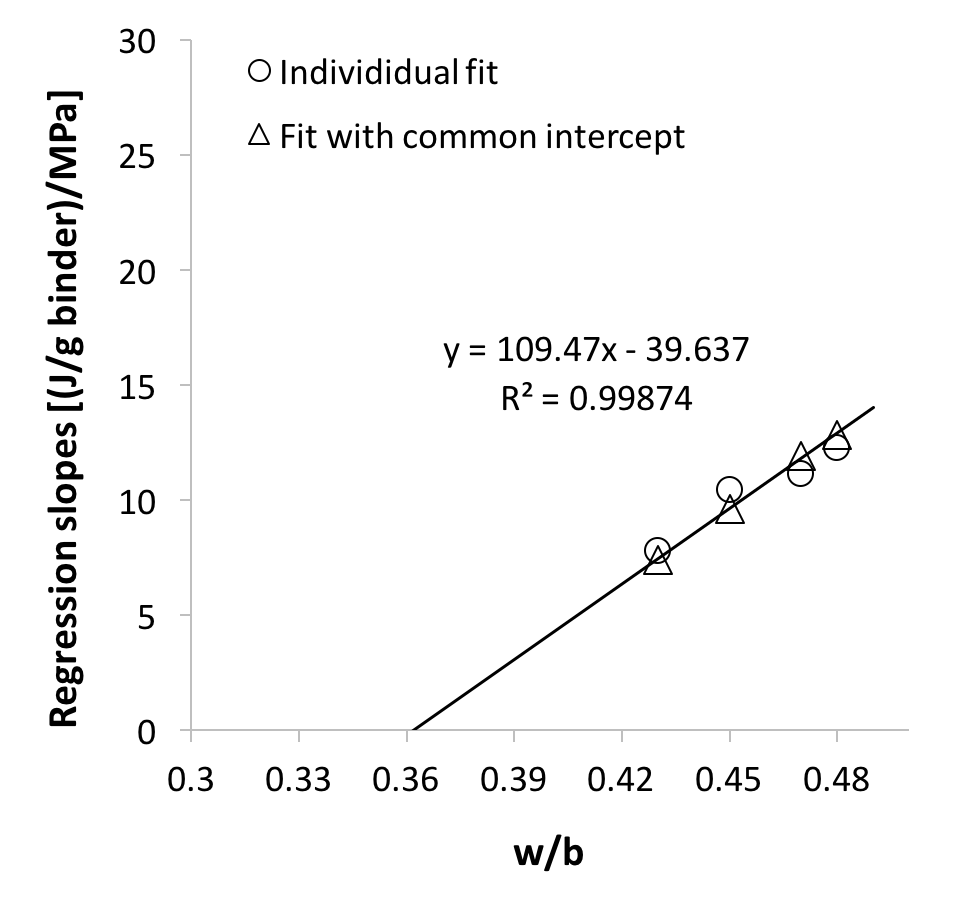 |
| --- | --- |

***Fig. S4*** *(corresponding to Fig. 5 in the paper) a) Ineffective heat* *H_0_*, *obtained from regressions in Fig. S3a, plotted versus w/b. As for Fig. 5a, no dependence on w/b ratio is observed. b) Regression coefficients from Fig. S3 versus w/b. The individual fit is obtained from Fig. S3a, while the one with the common intercept from Fig. S3b [2]*

| 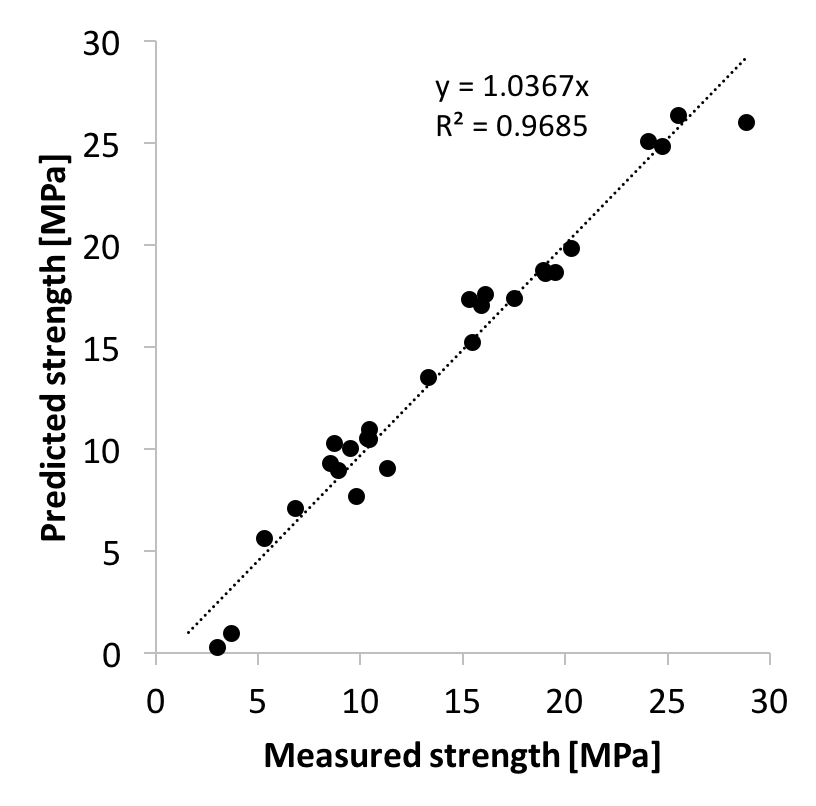 |
| --- |

***Fig. S5*** *(corresponding to Fig. 6 in the paper) Model for the prediction of compressive strength based on the measured compressive strength, as defined by [2], considering cumulative heat per gram of binder and including a dependence on the w/b*

[1] F. Boscaro, P. Juilland, L. Frunz, P. Kruspan, R. Flatt, Reproducibility of new low clinker concrete from the laboratory scale to the concrete plant, in: 1st International Conference on Innovation in Low-​Carbon Cement & Concrete Technology, London (UK), 2019.

[2] F. Boscaro, Research driven new low clinker activated blended cements, Doctoral Thesis, ETH Zurich, 2020. https://doi.org/10.3929/ethz-b-000447799.
